# Simulation Study of Surveillance Strategies for Faster Detection of Novel SARS-CoV-2 Variants

**DOI:** 10.3201/eid2911.230492

**Published:** 2023-11

**Authors:** Selina Patel, Fergus Cumming, Carl Mayers, André Charlett, Steven Riley

**Affiliations:** University College London, London, UK (S. Patel);; UK Health Security Agency, London (S. Patel, F. Cumming, C. Mayers, A. Charlett, S. Riley);; Imperial College London, London (S. Riley)

**Keywords:** COVID-19, simulation study, surveillance strategies, SARS-CoV-2, coronaviruses, viruses, novel virus variants, respiratory infections, detection, biosurveillance, travelers, zoonoses

## Abstract

Earlier global detection of novel SARS-CoV-2 variants gives governments more time to respond. However, few countries can implement timely national surveillance, resulting in gaps in monitoring. The United Kingdom implemented large-scale community and hospital surveillance, but experience suggests it might be faster to detect new variants through testing England arrivals for surveillance. We developed simulations of emergence and importation of novel variants with a range of infection hospitalization rates to the United Kingdom. We compared time taken to detect the variant though testing arrivals at England borders, hospital admissions, and the general community. We found that sampling 10%–50% of arrivals at England borders could confer a speed advantage of 3.5–6 weeks over existing community surveillance and 1.5–5 weeks (depending on infection hospitalization rates) over hospital testing. Directing limited global capacity for surveillance to highly connected ports could speed up global detection of novel SARS-CoV-2 variants.

In the current phase of the COVID-19 pandemic, waves of SARS-CoV-2 infection are driven by novel variants and their sublineages, which continue to cause illness and death with potential to disrupt society. Government policies to mitigate those effects are more effective if they are put in place early but have substantial associated costs and therefore should not be implemented unless necessary. Evaluating the threat of an emergent variant to determine a proportionate response requires time to gather evidence. Global surveillance of SARS-CoV-2 and other respiratory pathogen genome sequences aims to contribute to the rapid detection of novel variants so that countries have more time to make policy decisions to respond. However, few countries have the capacity and resources for timely national surveillance, resulting in gaps in international monitoring.

During the first few years of the pandemic, Hong Kong implemented a strict traveler quarantine protocol (*1*). Travelers underwent testing for SARS-CoV-2 infection during their quarantine, and 10% of detected imported infections were sequenced. Retrospective sequence data from those travelers reflects the global emergence and spread of variants over time. In some instances, traveler-based testing in Hong Kong detected variant circulation in other nations before it had been domestically sequenced and uploaded to GISAID (https://www.gisaid.org). The Hong Kong border screening experience suggests opportunities for traveler-based surveillance to speed up detection of novel variants and compensate for internationally incomplete coverage of domestic genomic surveillance.

To pilot this approach, the United States sampled arrival flights from countries with a high travel volume (India, South Africa, Nigeria, Brazil, France, United Kingdom, Germany) for voluntary surveillance testing (*2*). During November 2021‒January 2022, the United States achieved a 10% response rate and detected Omicron BA.2 seven days earlier and Omicron BA.3 forty-three days earlier than anywhere else in the country.

In the United Kingdom, although traveler-based surveillance was not used when border measures were decreased in 2022, previous traveler-based testing policies required inbound passengers to undergo testing shortly after arrival (*3*). The United Kingdom also conducted a large community survey of SARS-CoV-2 surveillance, and all patients experiencing symptomatic respiratory disease in hospital undergo testing for SARS-CoV-2 infection (*4*). Although reporting times were variable across those testing routes, Omicron was isolated and detected in England from a mandatory day 2 border test in an inbound traveler on November 16, 2021 (*5*). This test was 5 days earlier than a non–travel-associated sample that was obtained on November 21. Moreover, most of the earliest samples of Delta during the first 2 weeks of detection in the United Kingdom were also collected from travelers, despite the availability of universal testing in the community alongside surveillance at that time (*6*). To explore the potential utility of border screening for more rapid detection of variants, we simulated the time to obtaining a sample of an imported novel variant for genomic sequencing through sampling arrivals at ports in England, compared with existing large-scale community surveillance and testing of persons who came to a hospital. 

## Methods

Variants in our scenarios are considered to be imported from a country of a similar level of connectedness as between England and China. Over the most recent winter (December 2022–January 2023), China showed a huge increase in transmission of SARS-CoV-2 and resulting deaths after lifting of regulations that were part of previous Zero-COVID policy (*7*). This transmission risks the emergence of novel variants that could have a major effect on the epidemiology of COVID-19 elsewhere in the world. We replicated simulations for 4 scenarios of imported novel variants with infection hospitalization rates (IHRs) of 1.0%, 1.5%, 2.0%, and 2.5%. During the initial spread of the Alpha variant, the IHR was estimated at 1.0%–2.0%, which caused major impact and resulted in the reintroduction of national lockdown laws to mitigate its spread (*8**,**9*).

We generated a single-wave epidemic curve originating in an area with a total population of 60 million. The index case occurred on day 0. A Poisson distribution with a mean of 2 was assumed as the offspring distribution (i.e., each case, on average, transmits an infection to 2 other persons). The distribution of the generation time (the interval between the infection in a primary case and the infection in a secondary case caused by a transmission from the primary case) was assumed to be a gamma distribution with a shape parameter of 7 and a scale parameter of 1 Thus, the effective reproduction number was 2, and the average doubling time was 7 days. The offspring distribution for the first 2 generations was fixed at exactly 2. We assumed that the epidemic increased unchecked for 16 weeks, after which the mean of the offspring distribution was reduced to represent both control countermeasures and depletion of susceptible persons in the population. Between the 17th and 26th generations, we reduced the mean by 0.1 at each successive generation, such that the reproduction number was 1 at the 26th generation. From the 27th generation onward, the mean of the offspring distribution was reduced at each generation by 0.01786 (1/56).

The incubation period for each generated infection was drawn from the published pooled lognormal distribution in McAloon et al. (*10*). This procedure provides an estimated mean of 1.63 and SD of 0.5 for a normal distribution of the logged incubation period distribution. Published estimates of the infectious period before and after symptom onset are extremely heterogeneous, as described in Byrne et al. (*11*). Thus, the presymptomatic infectious period was fixed at 2 days, and the combined presymptom and postsymptom infectious period for each generated infection was drawn from a normal distribution with a mean of 10 days and an SD of 1.33 days. This procedure provides a relatively small probability of being infectious 10 days after symptom onset, as reported by Singanayagam et al. (*12*). We rounded those 2 periods to an integer, providing the duration for disease. Daily prevalence as estimated by combining the simulated cases over their duration for all days after the day the index case occurred. In the simulations, the period postinfectiousness in which PCRs could still detect virus was ignored. The simulated epidemic curve was truncated at 300 days.

We obtained the number of incoming travelers on each day that were incubating or infectious by using a draw from a binomial distribution. We assumed that the number of daily travelers was fixed at 250 and a probability equal to the origin areas prevalence on that day (i.e., assuming that persons infected are as equally likely to travel as persons not infected).

For detection at the border, conditional on the simulations having >1 infected traveler, we selected a representative sample ranging from 10% to 50% of travelers for testing. We further assumed that the percentage who are in an infectious state (detectable) was 73%, the sensitivity of the test 85%, and the percentage of positive test results, 50%. We used those percentages as the probability of draws from independent Bernoulli distributions; a detection was declared if each of those draws were 1.

We assumed growth in the destination country to be the same as growth in the origin area. Incubating or infectious incursions were drawn from a Bernoulli distribution with a probability of 73%. The time remaining in these states was obtained from a uniform distribution and the mean of the offspring distribution modified to account for this time. We assumed that travelers would spend all of their infectious period in the destination country. Daily incidence and prevalence of cases in the destination country were generated, but with the destination country population being assumed to be 56 million. We simulated 1,000 destination country epidemics.

For detection of a simulated case in the hospital setting, we assumed IHRs of 1.0%–2.5% and allocated simulated cases to presence in a hospital by using a draw from a Bernoulli distribution with a probability of 1%. We assumed that time to seeking care at a hospital because of infection followed a gamma distribution with a shape parameter of 1.4 and a scale parameter of 4 (i.e., giving a mean of 5.6 days, but with substantial variation). The percentage of persons seeking care who were tested was 50%; sensitivity of the test and percentage of positive test results sequenced were set as previously stated. Simulations were applied to each of the 1,000 destination country epidemics.

For detection of a simulated case in a community setting, we used a range of community cohort surveillance sizes from 20,000 (≈0.04% of the population) to 200,000 (≈0.36% of the population). We assumed that each person in this surveillance was tested every 2 weeks. We applied simulations to each of the 1,000 destination country epidemics The number detected each day obtained from a draw from a binomial distribution by using the number tested each day and the simulated daily prevalence, combined with the sensitivity of the test and the percentage of positive test results.

The time to detecting a case from border, hospital, and community testing has been summarized by using the empirical 5th, 25th, 50th, 75th, and 95th percentiles of the simulation sets. We ran simulations using Stata version 17.0 (StataCorp LLC, https://www.stata.com). For all simulation sets, we used a unique random number seed in a 64-bit Mersenne Twister pseudo-random number generator (default pseudo-random number generator in Stata). A detailed technical description of the methods used is available (Appendix 1).

## Results

First, we simulated the time to detection of an imported novel variant through different sampling fractions (10%, 20%, 30%, 40%, and 50%) of traveler arrivals in England. We assumed that the prevalence of infection in the passenger population was equal to that of the epidemic curve generated for the country of origin over time (Appendix 1). In our scenarios, there was a nonlinear relationship between increasing sampling fraction and decreasing days to detection starting from 131 days to detection through sampling 10% of passenger arrivals (Table 1). The greatest reduction in time to detection was gained between sampling fractions 10%‒20%, which led to a median 8-day decrease in time to detection. Thereafter, the time gained began to decrease with increasing sampling fraction.

**Table 1:** Simulated time to detect a novel variant since index case in study of traveler testing for surveillance of novel SARS-CoV-2 variants

Percentage tested	Empirical percentiles of simulated time to detection distribution, d				
	5th	25th	Median	75th	95th
10	104	121	131	140	150
20	96	114	123	131	141
30	94	110	119	126	136
40	89	107	115	123	131
50	86	105	114	121	130

Next, we simulated the time to detection through testing 50% of persons coming to a hospital in England. We assumed that growth in incidence in England (the destination country) was the same as that in the country of origin. We ran simulations for scenarios where variants had IHRs of 1.0%, 1.5%, 2.0%, and 2.5%. Although time to detection in hospitals decreased with increasing IHR, in all 4 scenarios it took >10 days longer to detect a novel variant in hospitals than by sampling 10%–50% of travelers arriving in England (Table 2).

**Table 2:** Simulated time to detect a novel variant since index case through hospital testing in study of traveler testing for surveillance of novel SARS-CoV-2 variants

Infection hospitalization rate (%)	Empirical percentiles of simulated time to detection distribution, d				
	5th	25th	Median	75th	95th
0.01 (1)	124	141	150	157	167
0.015 (1.5)	122	138	147	154	162
0.02 (2)	117	134	143	151	159
0.025 (2.5)	115	132	142	149	159

Finally, we simulated the earliest time to obtaining a sample of an imported novel variant through testing a community cohort sampled for surveillance. We ran scenarios implementing a sample size of 0.04% (20,000) to 0.36% (200,000) of the population in England, assuming the same growth in prevalence in the population over time as that assumed for incidence. Increasing the size of the community cohort from 0.04% to 0.36% of the population decreased the time to detection by 3 weeks (175 days reduced to 154 days) (Table 3). For the sample size of existing community surveillance in England, which comprises ≈140,000 tests every 2 weeks, the simulated earliest time to detection was 157 days.

**Table 3:** Simulated time to detect a novel variant since index case through community testing in study of traveler testing for surveillance of novel SARS-CoV-2 variants

Community testing cohort size (% destination country population)	Time to detection (days since emergence of index case), summaries from 1,000 simulations				
	5th percentile	25th percentile	Median	75th percentile	95th percentile
20,000 (0.04)	145	165	175	183	191
30,000 (0.05)	144	161	170	178	187
40,000 (0.07)	140	158	168	176	185
50,000 (0.09)	138.5	156	166	175	184
60,000 (0.11)	137	155.5	165	172	182
70,000 (0.13)	137	154	163	171	181
80,000 (0.14)	136	153	162	170	179
90,000 (0.16)	133	151	161	169	177
100,000 (0.18)	133.5	150.5	160	168	178
110,000 (0.20)	130	150	159	167	176
120,000 (0.21)	130	148	158	166.5	176
130,000 (0.23)	130.5	149	158	165	174
140,000 (0.25)	129	148	157	164	173
150,000 (0.27)	129	146	156	163	172
160,000 (0.29)	127.5	146	155.5	163	172
170,000 (0.30)	127	146	155	164	173
180.000 (0.32)	126	145	154	162	171
190,000 (0.34)	128	145	154	162	173
200,000 (0.36)	127	144.5	154	162	171

We found that, for border testing, the range of the median time to detection from the index case was 131 days (10% of travelers tested) to 114 days (50% of travelers tested). This result compares with 150 days (1% IHR) to 142 days (2.5% IHR) for the median of the earliest time to detection in hospitals, assuming 50% of persons seeking care are tested. Also, we found medians of 175 days (testing a cohort of 0.04% of the population) versus 154 days (testing a cohort of 0.36% of the population) for the earliest time to detection through community surveillance. Detailed study results are provided (Appendix 2).

## Discussion

Our simulations indicate that sampling a relatively small percentage, 10%, of inbound travelers for surveillance could reduce the time to detection of the first case of an imported novel variant of SARS-CoV-2 in the England by 26 days compared with existing community surveillance. Increasing sampling fraction of travelers to 50% could increase this speed advantage to 43 days. Depending on IHR (1.0%–2.5%), sampling 10% of inbound travelers would also detect a variant 11–19 days faster than testing hospital admissions for surveillance. However, sampling 50% of arrivals would lead to detection 4–5 weeks faster than hospital testing.

Our simulated results appear concordant with the closest available observed data. In the United States, testing 10% of passengers on arrival flights from countries with a high travel volume resulted in Omicron BA.2 being detected 7 days earlier and Omicron BA.3 being detected 43 days earlier than anywhere else in the country (*2*). In comparison with our scenarios, a 10% sampling fraction resulted in detection of a novel variant 1.5–4 weeks sooner than in other settings. However, the extent to which further comparisons can be drawn between our results and this experience is limited. The scale of community and healthcare surveillance in the United States is much smaller than is assumed in our scenarios, and, unlike in our scenarios, US arrivals were required to present a negative test result before departure. In addition, the time between specimen collection and reporting sequence data can be extremely variable between testing pathways, which makes it challenging to observe the speed advantage gained in this example through sampling strategy alone.

Our findings are also broadly in agreement with more distantly related retrospective data from community testing and policies such as managed quarantine services (MQS) and requirement to test on or shortly after arrival in a country. Testing inbound travelers has detected or collected some of the earliest samples of imported novel variants nationally and globally, even during periods when universal testing has been available in the community. In Hong Kong, sequence data were collected for 10% of all infections detected through MQS. Retrospective analysis of those records and external data sources indicate that traveler-based testing was either a good reflection, or an early indicator, of the global emergence and spread of novel variants. For example, Omicron (B1.1.529) was detected in Hong Kong through a sample obtained in an MQS on November 13, 2021 (*13*), which was uploaded to GISAID on November 23 (*13*). This upload triggered UK investigations on November 24, resulting in government intervention to delay further introduction and spread (*14*). Most of the earliest samples of Omicron subsequently collected in the United Kingdom were from persons who had recently traveled (*5*). Thus, Omicron samples collected through MQS in Hong Kong were able to be used as prospective evidence for policy decisions because of rapid genomic sequencing of samples and data reporting. In the United States, early samples of Omicron were also collected, frequently from persons who had a history of recent travel. However, long lag times from data collection to reporting indicated that this factor was not known until December 1, 2021 (*15*).

We also report that sampling 50% of persons seeking care at hospitals for surveillance in our scenarios detected a novel variant with an IHR of 2.5% ≈8 days faster than a variant with an IHR of 1.0%. A lower IHR could either be caused by less severe disease associated with the variant or the availability of effective COVID-19 therapies preventing severe outcomes. An increased number of persons seeking care at hospitals when IHR is greater reduces the speed advantage gained through traveler-based surveillance. However, waves of infection caused by variants that have higher IHRs are more likely to be detected earlier in the country of emergence as a result of increasing hospital visits. This factor often already offers governments outside the country of emergence some advanced warning of the impact of a new wave of infection associated with greater illness and death, despite gaps in global genomic surveillance. Therefore, the greatest potential impact of early detection through genomic surveillance might be for those variants that have an IHR large enough to cause societal disruption but low enough that it is slower to identify through hospital admissions.

To simulate the time to detection of an imported novel variant in England in each of our scenarios, we have made some simplifying assumptions. We have assumed that the prevalence of infection in air passengers is the same as that in the country of origin at the time of the departure of their flight, specimens are collected from a random sample of passengers, and the variant doubling time in the destination country is the same as that of in country of origin once seeded. A lower reproductive rate across both countries would have extended the time to detection of a novel variant across all surveillance strategies. However, a lower reproductive rate in only the destination country would have increased the speed advantage of border surveillance testing strategies.

We have also considered only direct incursions from the country of emergence of a novel variant to the destination country. We have not considered the effect of indirect incursions linked to infected travelers arriving from other countries where transmission might also be occurring. This decision is a simplification of observed human behavior, population immunity profiles, and transmission dynamics. However, we do not expect that a model comprising more complex representations of those processes would result in greatly different overall conclusions. We have also not attempted to carry out an economic evaluation of each surveillance strategy. Although such an evaluation is a major factor in policy and public health decisions, it would require a detailed cost-effectiveness analysis that is beyond the scope of this study.

In this report, we have focused the results and discussion on simulated scenarios that compare border surveillance with existing surveillance in hospitals and the community in England and the United Kingdom. However, this surveillance in England achieved greater coverage than for most countries. Therefore, as routine testing and surveillance for SARS-CoV-2 is decreasing globally, this study probably provides conservative estimates of the potential speed advantage that could be gained through traveler-based surveillance approaches. Also, if there were concerns about a specific country at any point in time, temporary programs would be able to achieve high sample proportions at the border with only limited numbers of samples compared with other ongoing or potential global programs.

It is useful to recognize that the collection of a sample of a novel variant for detection is the first step to evaluate the threat of a novel variant. In our scenarios, we do not consider the time it takes to sequence and report data obtained from a sample. Sequencing and reporting times are extremely variable across countries which can greatly reduce the time gained through effective sampling approaches (*16*). In addition, a full threat assessment requires robust estimates of severe outcomes in addition to temporal and geospatial descriptions of variant epidemiology to inform policy decisions.

Global surveillance of SARS-CoV-2 genome sequences contributes to rapid detection of novel variants to give governments more time to respond. However, few countries have capacity to implement national surveillance with timely sequencing and reporting, resulting in major gaps in global coverage of surveillance. In our scenarios, directing limited global capacity for surveillance to the most highly connected ports could provide governments with much more time to respond to future novel variants of SARS-CoV-2 and their sublineages. Beyond informing national approaches to surveillance, this approach also underscores the potential usefulness of international collaboration to achieve high global coverage of surveillance and provide governments with more time to make policy decisions to respond to novel variants of SARS-CoV-2.

Appendix 1 Additional information on simulation study of surveillance strategies for faster detection of novel SARS-CoV-2 variants showing methods for earliest detection of a novel variant by border testing, hospital, and community surveillance.

Appendix 2 Additional information on simulation study of surveillance strategies for faster detection of novel SARS-CoV-2 variants showing results of simulations.
