# Supplementary material for: Simulation Study of Surveillance Strategies for Faster Detection of Novel SARS-CoV-2 Variants
Source: Emerg Infect Dis. 2023 Nov;29(11):2292–7. doi: 10.3201/eid2911.230492 (PMC10617356; doi:10.3201/eid2911.230492)
Supplement: Appendix 1 — Additional information on simulation study of surveillance strategies for faster detection of novel SARS-CoV-2 variants showing methods for earliest detection of a novel variant by border testing, hospital, and community surveillance. [file 23-0492-Techapp-s1.pdf]

Article DOI: https://doi.org/10.3201/eid2911.230492

EID cannot ensure accessibility for Supplemental Materials supplied by authors. Readers who have difficulty accessing supplementary content should contact the authors for assistance.

Simulation Study of Surveillance Strategies for Faster Detection of Novel SARS-CoV-2 Variants

Appendix 1

Methods for earliest detection a novel variant by border testing, hospital, and community surveillance

Simulated epidemic at origin

A single occurrence of a novel variant occurs on day zero (d_0). To obtain the resultant epidemic curve, offspring, and generation time, distributions of $Poisson(2)$ and $\Gamma(7,1)$ were used respectively with the offspring fixed at 2 for the first 2 generations to ensure the epidemic establishes (Appendix 1 figure 1). Random draws of generation times were rounded to the nearest integer.

This choice of offspring and generation time provides an epidemic curve where R_0 is 2 with a doubling time of 7 days. The variant was assumed to grow unchecked for 16 generations, after which the mean of the offspring distribution was reduced by 0.1 of each successive generation to obtain an offspring distribution $Poisson(1)$ at the 26th generation, i.e., R_t of 1 after around 6 months. From the 27th generation onwards, the mean of the offspring distribution was reduced at each generation by 0.01786 (1/56).

Fifty-two generations were simulated, but a 300-day cut off used to ensure that chains with shorter than average generation times did not impact completeness of simulated infections, there being a probability of around 0.0002 of obtaining a sum of 52 draws from $round(\Gamma(7,1), 1)$ being <300.

For each simulated infection k, the day of infection d_k was obtained by summation of the generation times for their predecessors. The incidence on day d is obtained by a summation over all n infections, $IO_d = \sum_{k=1}^{n}(d_k == d)$, where $(d_k == d)$ is 1 if d_k equals d, 0 otherwise. The simulated epidemic curve is shown in Appendix 1 Figure 2, for which the cumulative incidence up to day 300 since d_0 is around 23,000,000.

For each simulated infection, a log incubation period was obtained from a random draw from $N(1.63, 0.5)$, with time of symptom onset occurring at $d_k^s = d_k + l_k$. The 10th, 50th, and 90th centiles for this incubation period distribution are ≈2.7, 5.1, and 9.7 days, respectively (Appendix 1 Figure 3) (*1*).

The infectious period distribution was assumed to be $N(10, 1.33)$, with the period of infectiousness beginning 2 days before symptom onset. Thus, the first day of being infectious for the kth infection occurs on day $d_k^s - 2$. If $d_k^s - 2 < d_k$ then l_k is set to 0, and if $d_k^s - 2 > 19$ then l_k is set to 19. Appendix 1 Figure 3 provides a visualization of the assumed incubation period distribution and the temporal probability of infectiousness (*2,3*).

The pre-infectious period (d_k to $d_k^s - 3$) and infectious period ($d_k^s - 2$ to $d_k^s - 2 + i_k$) are combined and the prevalence of being in either state on day d is obtained from $PO_d = \frac{1}{N_o}\sum_{k=1}^{n}\left(d_k \leq d \leq (d_k + l_k + i_k - 2)\right)$, where $d_k \leq d \leq (d_k + l_k + i_k - 2)$ is 1 if d is greater or equal to $d_k$and less than or equal to $d_k + l_k + i_k - 2$, 0 otherwise. The infection prevalence is shown in Appendix 1 Figure 4.

Simulated epidemics at destination

For each day from d_0 it is assumed for simplicity, that there are a fixed number of direct air travelers n_t departing the origin for the destination. These travelers are assumed to have the same infection prevalence as the origin on the day of departure and all flights depart and arrive on the same day. On day d the number of infected travelers is obtained from a random draw from $binomial(n_t, PO_d)$. A simulated infected traveler is in the infectious state if a random draw from $binomial(1, \pi_{inf})$ is equal to 1, otherwise they are in a pre-infectious state. For the simulated incursions in both the pre-infectious and infectious state, the time in their state has been reduced by multiplication with a random draw from a uniform distribution

$Uniform([0,1])$, and for those in the infectious state, their simulated number of offspring has similarly been reduced.

One thousand simulated incidence growth curves have been generated from the simulated incursions, for a total of 30 generations. For simplicity, it is assumed that the offspring distribution and generation time distribution are the same as at the origin. While this is likely to be true for the latter, for the former this would implicitly assume that population mixing in the origin and destination are similar. It has also been assumed that detection is only possible during the infectious state, and the post infectiousness period where PCR tests could still detect virus has been ignored. Thus, $ID_d = \sum_{k=1}^{n}(d_k^s == d)$, where $(d_k^s == d)$ is 1 if d_k^s equals d, 0 otherwise.

The incidence growth curves have been converted to disease prevalence growth curves by using the same methods previously described but ignoring the days in the pre-infectious state. These are shown in Appendix Figures 5 and 6.

The disease prevalence on day d is obtained from $PD_d = \frac{1}{N_D}\sum_{k=1}^{n}\left(d_k^s - 2 \leq d \leq (d_k^s - 2 + i_k)\right)$, where $d_k^s - 2 \leq d \leq (d_k^s - 2 + i_k)$ is 1 if d is greater or equal to $d_k^s - 2$ and less than or equal to $d_k^s - 2 + i_k$, 0 otherwise.

While for most simulations the growth of incident cases is modest, there are a small proportion where exceptional growth is observed. Because the objective was to gain an understanding of the earliest time to detection, no account has been taken of either depletion of those susceptible in the population or effective control measures both of which would cause the simulations with exceptional growth to turn over and decline. It has been assumed that such behavior would only occur post the earliest detections so are not a major consideration.

Time to earliest detection

For each of testing arrivals at the border, testing those sufficiently ill to present at a hospital, and testing those enrolled in community surveillance, simulations were implemented as outlined in future sections with one thousand such simulations performed for each. For all simulation sets, a unique random-number seed was used in a 64-bit Mersenne Twister pseudo-random-number generator.

The simulated earliest time of detection, i.e., earliest specimen date, ignoring any sample processing and reporting delays is obtained for each simulation with selected centiles of the simulated distributions of earliest detection times presented. For results in the paper, the median time to earliest detection is presented, with time in relation to the occurrence of the very first case d_0 being used throughout.

Time to earliest detection for testing at the border (T_b)

The number of incoming travelers n_d^I on each day that are either incubating or infectious is obtained by using a draw from $binomial(n_t, PO_d)$. For simplicity, an assumption that those traveling are independent of infection status has been made. Although this assumption will influence the time to earliest detection, which would occur later if prevalence in travelers is lower, it is unlikely to affect greatly any relative difference in the times to earliest detection at the border, or in either hospital or community surveillance.

The number of infected travelers being tested on day d, n_d^{tested} is obtained by using a draw from $binomial(n_d^I, \pi_t)$, provided n_d^I>0. The test on the kth simulated infection is considered positive (b_k^+) if it is an infectious state, not having a false negative test, and being successfully sequenced. This was obtained by multiplying the random draws from three Bernoulli distributions, $binomial(1, \pi_{inf})$, $binomial(1, \pi_{sens})$, and $binomial(1, \pi_{seq})$, with a detection being declared if each of these draws are 1, otherwise considered a failure to detect. It has been assumed that the microbiological test has a sensitivity of 85%, and specificity of 100%, and that for technical reasons only 50% of positives isolates will lead to a sequence being successfully obtained.

For each detection at the border b_k^+, $d_k^{b^+}$ is the day on which the positive specimen is taken. The time to earliest detection is $T_b = \min_{k \in \{b_k^+\}} d_k^{b^+}$.

Time to earliest detection for testing at hospitals (T_h)

From each of the 1000 simulated disease incidence growth curves in the destination country, the number of infections each day that would result in a hospital admission n_d^h was obtained from a random draw from $binomial(ID_d, \pi_{IHR})$, provided ID_d>0. The number of those hospitalized that will get tested, $n_d^{h^t}$ was obtained from a random draw from $binomial(n_d^h, \pi_h)$, provided n_d^h>0.

The kth infected simulation is considered positive h_k^+ if it is not a false-negative test result and is successfully sequenced. This was obtained by multiplying random draws from two Bernoulli distributions, $binomial(1, \pi_{sens})$, and $binomial\left(1, \pi_{seq}\right)$, with a detection being declared if each of both these draws are 1, otherwise considered a failure to detect.

For each hospital detection h_k^+, the day on which the positive specimen is taken $d_k^{h^+}$ was obtained from $d_k^s + t_h$, where d_k^s is the day of symptom onset of the kth infection, and t_h is the time from symptom onset to hospitalization, taken as a random draw from a $\Gamma(5,2)$ and rounded to the nearest integer as shown in Appendix 1 Figure 7. The time to earliest detection is obtained by using $T_h = \min_{k \in \{h_k^+\}} d_k^{h^+}$.

Time to earliest detection for testing in the community (T_c)

It has been assumed that persons in community surveillance test every 2 weeks, and for simplicity the number of daily tests is $\frac{n_c}{14}$. From each of the 1000 simulated disease prevalence growth curves in the destination country, the number of simulated prevalent infections that would be tested on day d in community surveillance $n_d^{c^t}$ was obtained from a random draw from $binomial\left(round\left(\frac{n_c}{14}, 1\right), PD_d\right)$, provided PD_d>0.

The kth infected simulation is considered positive c_k^+ if it is not a false negative test and is successfully sequenced. As before, this was obtained by multiplying random draws from two Bernoulli distributions, $binomial(1, \pi_{sens})$, and $binomial\left(1, \pi_{seq}\right)$, with a detection being declared if each of these draws are 1, otherwise considered a failure to detect. For each detection in the community c_k^+, $d_k^{c^+}$ is the day on which the positive specimen is taken. The time to earliest detection is obtained by using $T_c = \min_{k \in \{c_k^+\}} d_k^{c^+}$.

References

1. McAloon C, Collins Á, Hunt K, Barber A, Byrne AW, Butler F, et al. Incubation period of COVID-19: a rapid systematic review and meta-analysis of observational research. BMJ Open. 2020;10:e039652. PubMed https://doi.org/10.1136/bmjopen-2020-039652

2. Byrne AW, McEvoy D, Collins AB, Hunt K, Casey M, Barber A, et al. Inferred duration of infectious period of SARS-CoV-2: rapid scoping review and analysis of available evidence for asymptomatic and symptomatic COVID-19 cases. BMJ Open. 2020;10:e039856. PubMed https://doi.org/10.1136/bmjopen-2020-039856

3. Singanayagam A, Patel M, Charlett A, Lopez Bernal J, Saliba V, Ellis J, et al. Duration of infectiousness and correlation with RT-PCR cycle threshold values in cases of COVID-19, England, January to May 2020. Euro Surveill. 2020;25:2001483. PubMed https://doi.org/10.2807/1560-7917.ES.2020.25.32.2001483

Appendix1 Table. Parameters used

	Symbol	Estimates/distributions used
Population of origin	N_O	60,000,000
Population of destination	N_D	56,000,000
Offspring	c_k	$\mathrm{Poisson}(2)$
Generation time	g_k	$\mathrm{round}(\Gamma(7,1), 1)$
Incubation period	l_k	$\mathrm{round}(e^{N(1.63,0.5)}, 1)$
Infectiousness period	i_k	$\mathrm{round}(N(10,1.33), 1)$
Day of infection for the kth simulated infection	d_k	
Day of symptom onset for the kth simulated infection	d_k^s	
Infection incidence in origin on day d	IO_d	
Disease incidence in destination on day d	ID_d	
Infection prevalence in origin on day d	PO_d	
Disease prevalence in destination on day d	PD_d	
Direct travelers per day	n_t	100, 250, 500
Proportion of travelers tested	π_a	0.01, 0.02, 0.05.0.10, 0.20
Probability infectious $\overline{\iota_k/(l_k + \iota_k - 2)}$	π_{inf}	0.73
PCR Test sensitivity	π_{sens}	0.85
Positive successfully sequenced	π_{seq}	0.5
IHR	π_{IHR}	0.005,0.01,0.015,0.02,0.025
Proportion of hospital presentations testing	π_h	0.1, 0.2, 0.3, 0.4, 0.5
Time to hospital presentation	t_h	$\mathrm{round}((\Gamma(5,2), 1)$
Size of community surveillance	n_c	20,000–200,000 (steps of 10,000)

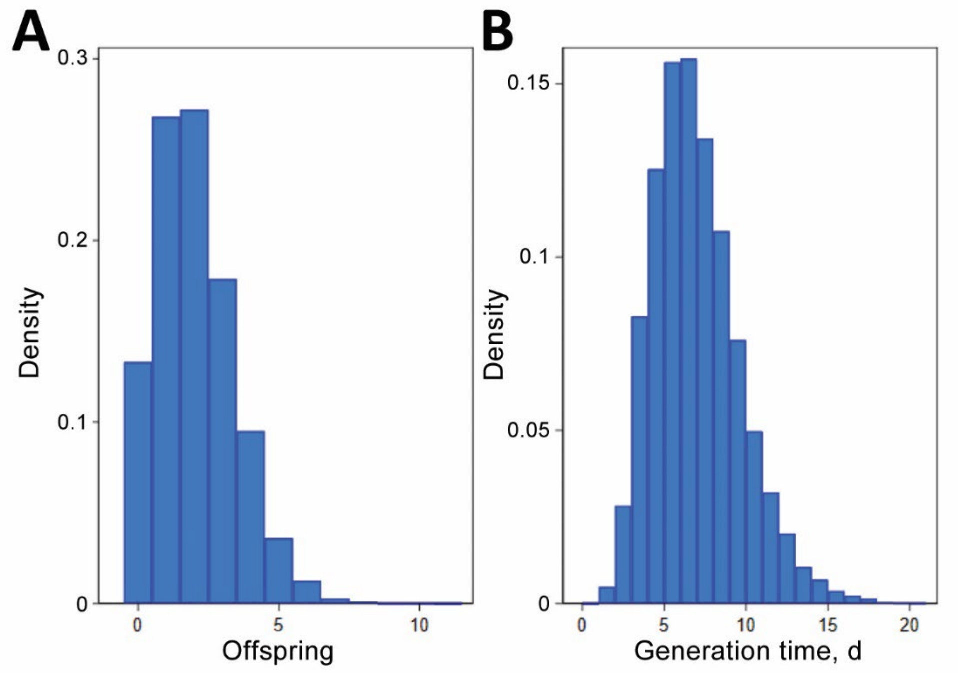

Appendix 1 Figure 1. Offspring (A) and generation time (B) distributions.

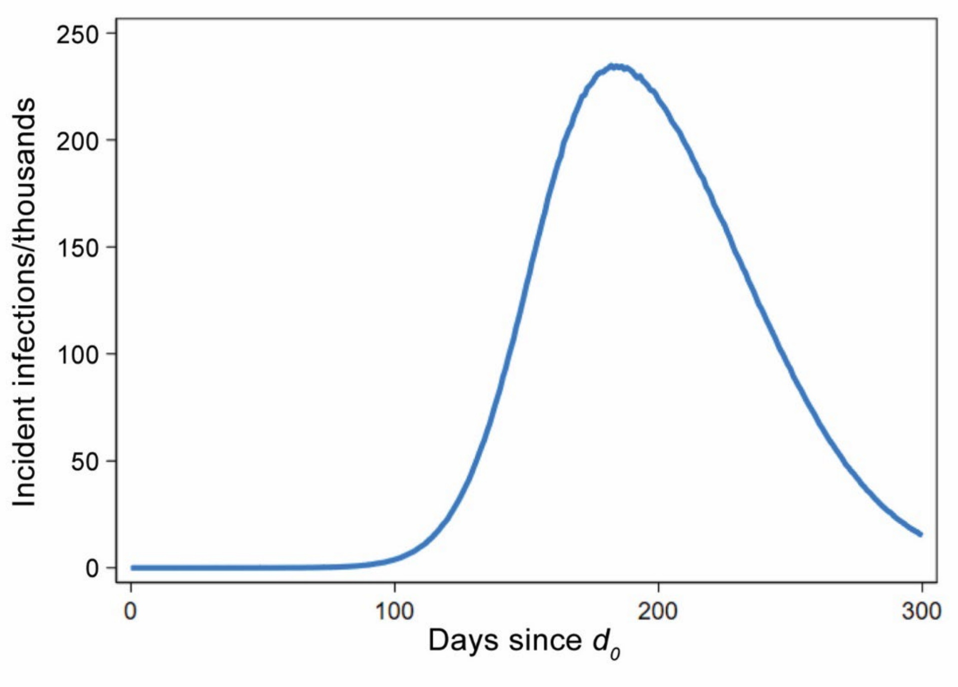

Appendix 1 Figure 2. Simulated epidemic curve at the origin.

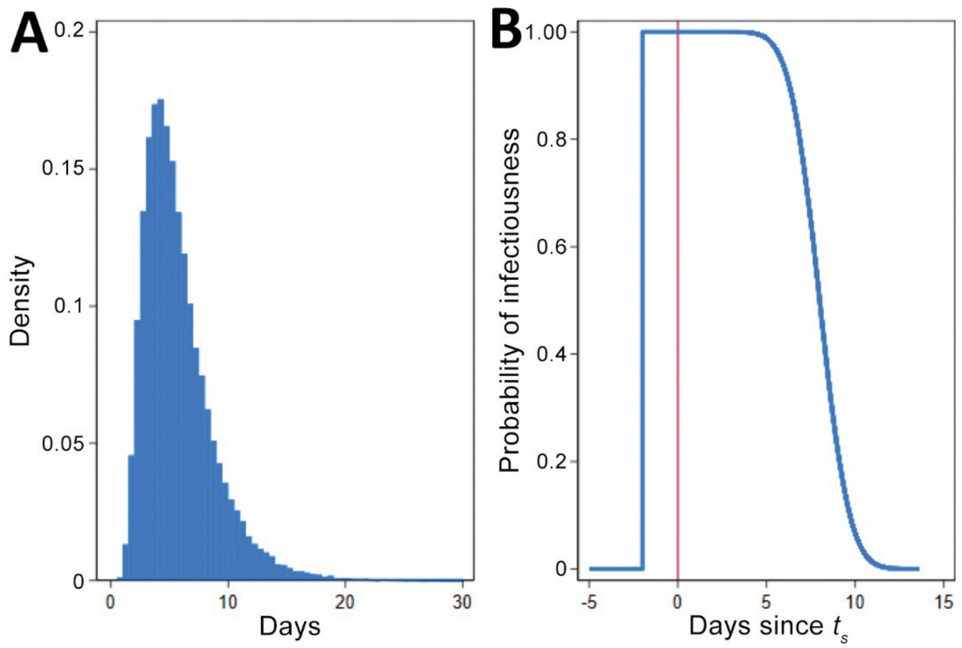

Appendix 1 Figure 3. incubation period distribution (A) and the probability of infectiousness (B).

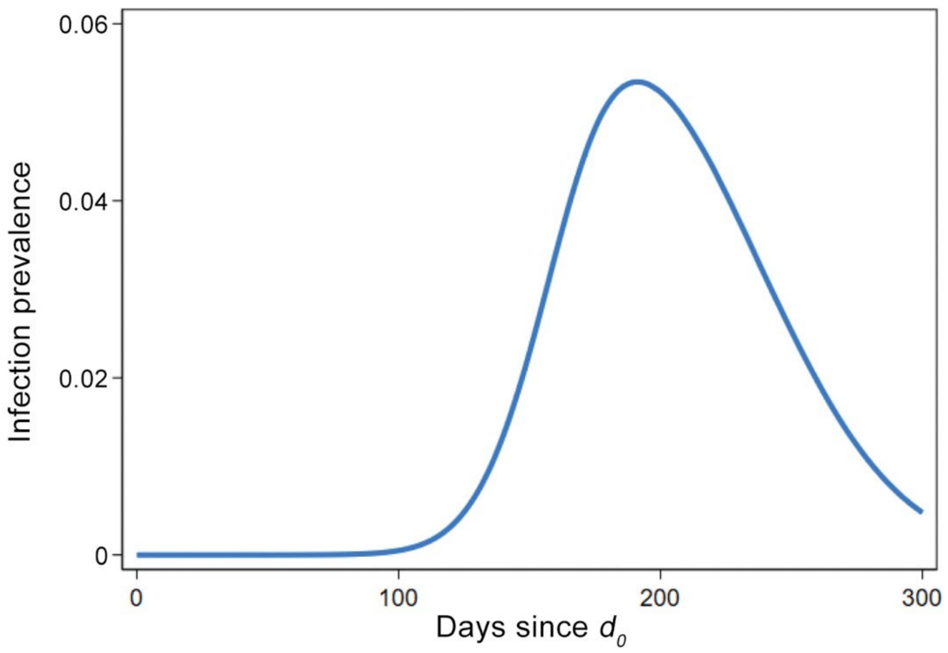

Appendix 1. Figure 4. Simulated infection prevalence at the origin.

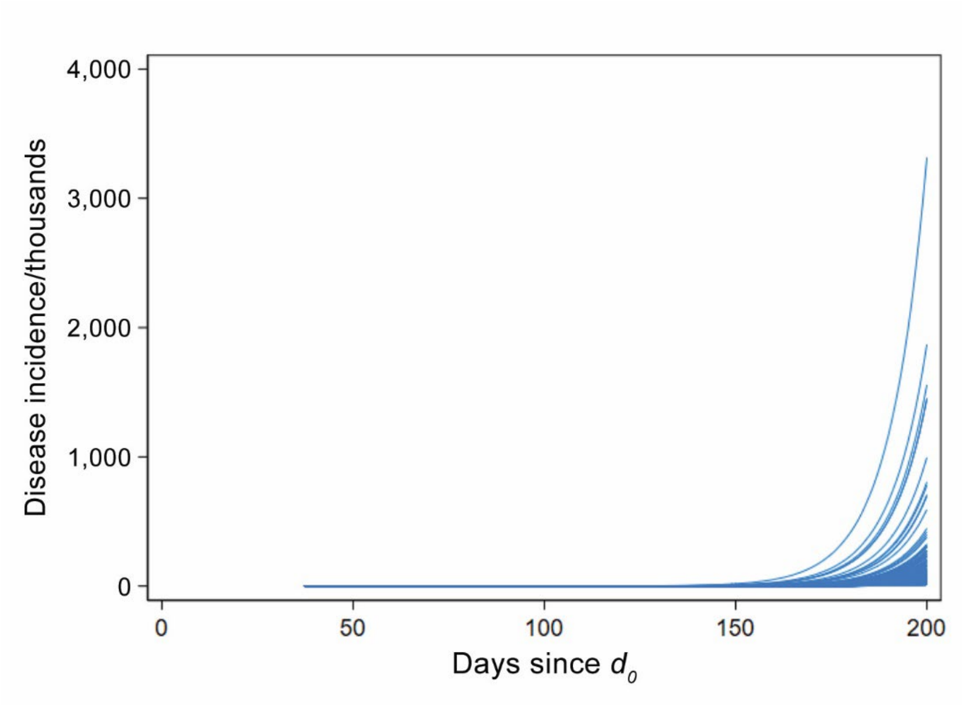

Appendix 1 Figure 5. Simulated disease incidence growth curves within the destination country for 500 arrivals per day by using daily prevalence from Appendix Figure 4.

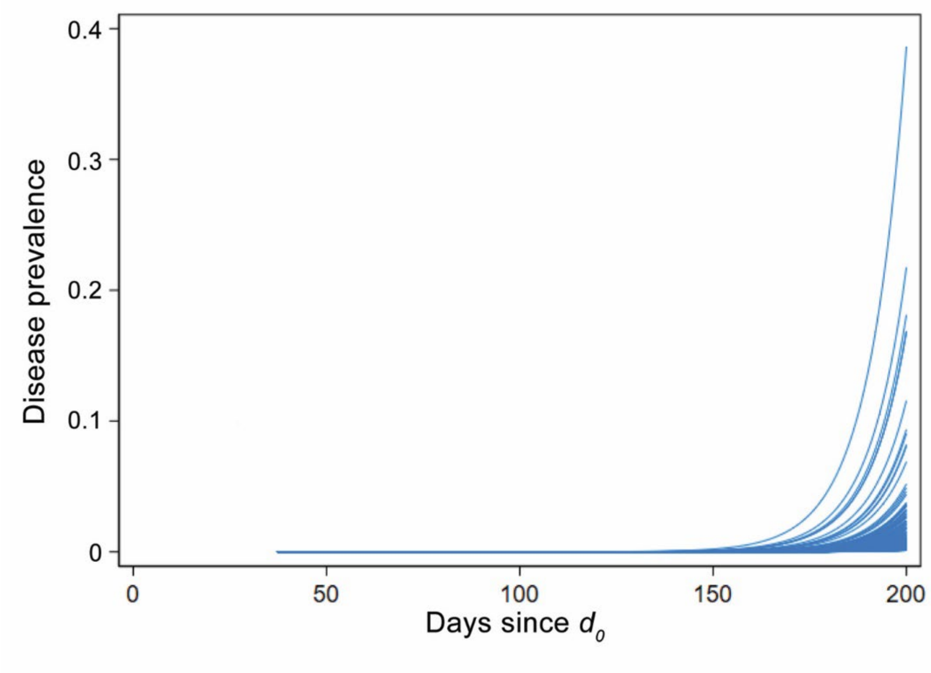

Appendix 1 Figure 6. Simulated disease prevalence growth curves within the destination country for 500 arrivals per day.

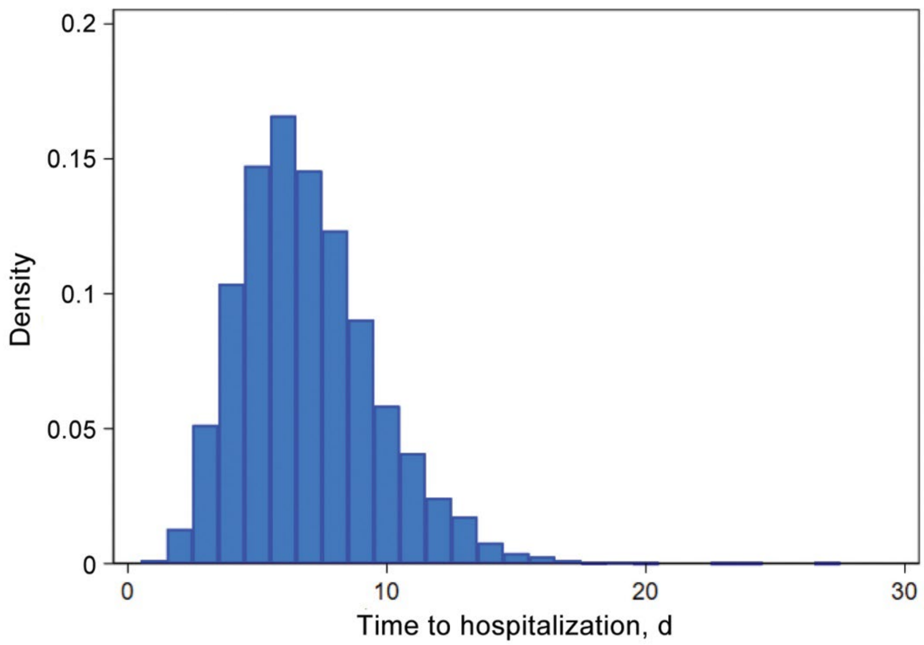

Appendix 1 Figure 7. Time from symptom onset to hospitalization distribution.
