# Supplementary material for: Simulation Study of Surveillance Strategies for Faster Detection of Novel SARS-CoV-2 Variants
Source: Emerg Infect Dis. 2023 Nov;29(11):2292–7. doi: 10.3201/eid2911.230492 (PMC10617356; doi:10.3201/eid2911.230492)
Supplement: Appendix 2 — Additional information on simulation study of surveillance strategies for faster detection of novel SARS-CoV-2 variants showing results of simulations. [file 23-0492-Techapp-s2.pdf]

Article DOI: https://doi.org/10.3201/eid2911.230492

EID cannot ensure accessibility for Supplemental Materials supplied by authors. Readers who have difficulty accessing supplementary content should contact the authors for assistance.

Simulation Study of Surveillance Strategies for Faster Detection of Novel SARS-CoV-2 Variants

Appendix 2.

Results of Simulations

The 8 tables below provide the results of the simulations described in the main paper and Appendix 1. The nature of simulations means that each time they are run, there will be slight variation in the results produced (not significant enough to change the trends or overall conclusions reported).

Appendix 2 Table 1. Summary statistics for the simulated earliest time to detection distribution for testing at the border with 250 daily passengers, all times refer to days since the index case

Proportion	centiles								
tested	1st	5th	10th	25th	50th	75th	90th	95th	99th
0.010	115	127	136	150	166	184	206	221	253
0.020	106	120	127	140	152	168	181	189	206
0.030	98	112	121	133	146	158	169	174	189
0.040	95	108	118	131	143	154	162	167	178
0.050	91	111	118	128	139	149	157	162	175
0.060	95	107	116	125	137	146	155	161	171
0.070	90	109	116	125	135	144	152	156	164
0.080	94	106	114	124	135	143	151	155	162
0.090	92	106	112	121	131	140	149	153	160
0.100	90	104	109	121	131	140	147	150	157
0.110	89	100	109	120	130	139	146	150	158
0.120	88	101	109	119	128	137	143	147	156
0.130	88	102	108	119	128	136	143	148	153
0.140	86	96	105	116	127	135	141	144	151
0.150	80	99	106	116	126	135	141	145	151
0.160	81	99	105	116	126	134	141	144	151
0.170	84	98	106	115	123	132	140	144	148
0.180	80	97	105	115	124	132	138	142	149
0.190	79	98	106	115	123	132	138	141	147
0.200	85	96	104	114	123	131	137	141	147
0.210	80	97	104	113	123	130	137	140	146
0.220	81	96	102	112	122	129	135	139	145
0.230	78	96	103	111	121	129	135	138	144
0.240	76	92	100	110	120	128	135	138	145
0.250	80	94	100	110	120	128	134	137	143
0.260	82	97	101	110	120	126	133	136	142
0.270	76	95	102	110	119	127	133	137	144
0.280	78	95	101	111	119	126	132	136	142
0.290	73	92	101	109	118	126	132	136	142
0.300	83	94	100	110	119	126	132	136	142

Proportion tested	centiles								
	1st	5th	10th	25th	50th	75th	90th	95th	99th
0.310	76	94	100	109	118	126	131	135	141
0.320	70	90	98	109	117	125	131	134	140
0.330	72	89	97	108	117	125	131	135	140
0.340	77	92	98	106	117	124	130	133	138
0.350	75	89	97	108	116	124	130	134	139
0.360	72	91	98	107	116	124	129	133	140
0.370	65	90	97	106	115	123	130	133	137
0.380	78	91	97	107	116	124	129	133	138
0.390	66	91	97	106	115	122	129	132	138
0.400	78	89	96	107	115	123	128	131	138
0.410	78	90	97	105	115	123	128	131	138
0.420	76	90	97	106	115	122	128	131	136
0.430	74	86	96	107	115	122	128	131	137
0.440	77	90	98	107	115	122	128	130	135
0.450	65	87	95	106	115	122	127	132	138
0.460	68	88	94	105	114	121	126	129	136
0.470	75	90	96	105	113	120	127	129	135
0.480	78	90	96	104	113	120	126	130	135
0.490	73	88	95	105	114	121	126	129	134
0.500	72	86	94	105	114	121	126	130	134

Appendix 2 Table 2. Summary statistics for the simulated earliest time to detection distribution for testing at hospitals with seedings from 250 daily passengers

IHR	Proportion tested	centiles								
		1st	5th	10th	25th	50th	75th	90th	95th	99th
0.005	0.10	128	143	150	161	171	178	185	188	195
0.005	0.20	119	139	145	155	164	173	179	181	187
0.005	0.30	114	134	140	151	161	168	175	178	183
0.005	0.40	116	132	140	150	159	166	172	175	181
0.005	0.50	117	128	137	146	156	164	169	173	179
0.010	0.10	123	137	144	155	165	172	178	182	187
0.010	0.20	115	131	137	147	157	165	172	175	181
0.010	0.30	115	129	136	145	154	162	169	172	178
0.010	0.40	108	124	131	141	151	159	165	168	173
0.010	0.50	107	124	132	141	150	157	164	167	170
0.015	0.10	112	133	139	151	161	168	174	178	183
0.015	0.20	108	125	133	144	155	162	169	172	177
0.015	0.30	101	122	129	142	152	159	165	168	174
0.015	0.40	104	120	128	139	147	156	162	165	169
0.015	0.50	108	122	128	138	147	154	159	162	167
0.020	0.10	115	132	138	149	158	166	171	174	181
0.020	0.20	108	123	131	142	152	160	166	168	174
0.020	0.30	110	123	128	138	148	156	161	165	172
0.020	0.40	102	119	126	137	146	154	160	163	166
0.020	0.50	103	117	124	134	143	151	156	159	164
0.025	0.10	111	128	135	147	156	163	169	173	177
0.025	0.20	108	122	129	140	149	157	162	166	172
0.025	0.30	104	118	127	138	147	154	159	162	168
0.025	0.40	104	119	125	135	143	151	157	159	164
0.025	0.50	104	115	122	132	142	149	155	159	163

Appendix 2 Table 3. Summary statistics for the simulated earliest time to detection distribution for testing at the border with 500 daily passengers, all times refer to days since the index case

Proportion	centiles								
tested	1st	5th	10th	25th	50th	75th	90th	95th	99th
0.010	108	124	130	142	154	167	179	186	207
0.020	103	114	122	131	143	153	163	170	181
0.030	94	109	116	127	138	147	156	160	172
0.040	93	106	112	122	135	144	152	156	163
0.050	89	103	111	122	131	140	147	151	161
0.060	87	102	109	118	129	136	143	146	155
0.070	88	100	108	118	127	136	142	146	154
0.080	79	98	106	116	125	133	140	143	149
0.090	84	97	104	114	124	132	138	142	147
0.100	82	96	103	114	124	132	137	140	147
0.110	81	95	103	112	122	130	136	140	146
0.120	79	94	103	112	121	129	136	138	143
0.130	75	93	102	111	120	128	133	137	143
0.140	77	93	100	110	119	127	133	137	140
0.150	79	93	99	109	118	126	132	135	140
0.160	75	92	98	108	118	125	132	136	140
0.170	79	93	99	109	118	125	131	134	140
0.180	73	89	97	108	116	124	130	133	138
0.190	73	88	97	107	115	123	129	133	138
0.200	76	91	97	105	114	122	127	131	137
0.210	79	90	97	106	115	123	129	131	137
0.220	74	92	97	106	114	121	127	130	137
0.230	76	89	96	106	114	121	127	130	136
0.240	72	87	93	104	113	121	126	130	134
0.250	73	88	97	105	113	120	126	129	135
0.260	68	89	95	105	113	120	126	129	133
0.270	71	87	93	103	112	119	125	127	132
0.280	70	87	95	104	112	119	124	128	134
0.290	64	85	92	101	111	119	125	128	133
0.300	65	83	91	102	111	118	125	127	132
0.310	70	85	92	103	111	118	123	126	131
0.320	70	86	92	102	111	117	123	125	130
0.330	68	86	92	101	110	117	124	127	131
0.340	71	84	90	101	110	117	123	126	131
0.350	71	87	93	101	111	117	123	125	131
0.360	69	86	92	100	109	116	121	125	130
0.370	68	84	91	101	109	116	122	125	129
0.380	68	84	92	100	109	116	121	124	127
0.390	68	84	89	101	109	116	120	123	128
0.400	71	84	90	100	109	116	121	123	128
0.410	65	84	90	100	108	115	120	123	127
0.420	71	86	91	100	107	114	120	122	127
0.430	72	84	90	99	108	115	121	123	128
0.440	69	82	88	98	108	115	120	123	129
0.450	68	82	88	99	107	114	119	122	127
0.460	65	81	88	98	107	115	120	123	128
0.470	56	82	87	98	106	114	119	122	127
0.480	61	83	89	98	106	113	118	121	127
0.490	63	81	88	98	106	113	119	121	125
0.500	63	79	87	97	106	113	118	121	127

Appendix 2 Table 4. Summary statistics for the simulated earliest time to detection distribution for testing at hospitals with seedings from 500 daily passengers

IHR	Proportion tested	centiles								
		1st	5th	10th	25th	50th	75th	90th	95th	99th
0.005	0.10	115	135	143	154	163	171	177	179	184
0.005	0.20	114	128	135	147	156	164	169	172	178
0.005	0.30	117	127	133	144	153	161	167	169	173
0.005	0.40	108	122	131	141	149	157	163	166	172
0.005	0.50	109	121	130	140	148	156	161	164	169
0.010	0.10	121	132	138	147	156	164	170	174	177
0.010	0.20	109	123	128	140	149	157	163	167	172
0.010	0.30	104	121	128	137	147	154	160	163	167
0.010	0.40	105	119	125	135	144	151	157	160	167
0.010	0.50	98	116	123	134	142	149	155	158	163
0.015	0.10	110	128	133	144	153	160	165	168	172
0.015	0.20	104	120	126	137	147	154	160	162	169
0.015	0.30	103	118	125	134	143	151	156	159	164
0.015	0.40	100	112	121	132	141	148	154	157	162
0.015	0.50	95	112	119	130	139	145	151	154	158
0.020	0.10	107	122	130	140	150	158	163	166	172
0.020	0.20	106	118	125	135	144	152	157	161	166
0.020	0.30	101	115	121	132	141	148	153	156	163
0.020	0.40	104	114	119	129	139	146	151	154	159
0.020	0.50	96	111	117	127	136	143	149	151	158
0.025	0.10	107	121	130	139	148	155	162	164	169
0.025	0.20	105	116	123	133	143	150	155	158	162
0.025	0.30	95	114	120	130	138	146	151	154	159
0.025	0.40	94	111	117	127	137	143	149	152	157
0.025	0.50	96	108	115	125	134	141	147	150	154

Appendix 2 Table 5. Summary statistics for the simulated earliest time to detection distribution for testing in community surveillance with seedings from 500 daily passengers

Community survey size	centiles								
	1st	5th	10th	25th	50th	75th	90th	95th	99th
20000	125	139	147	157	166	174	179	183	189
30000	122	137	144	153	163	171	177	180	184
40000	124	134	141	152	161	167	173	176	181
50000	118	132	139	148	158	166	171	173	180
60000	117	131	137	147	157	164	170	173	178
70000	114	127	134	145	155	162	168	171	178
80000	115	128	135	144	154	161	167	170	176
90000	113	126	133	144	153	161	166	169	173
100000	112	128	133	144	152	160	165	168	173
110000	107	126	131	142	152	158	164	167	172
120000	106	123	130	140	150	158	163	167	172
130000	107	123	131	141	150	157	164	167	171
140000	109	124	130	139	148	156	162	164	169
150000	106	122	129	139	148	156	161	164	170
160000	107	120	128	139	148	155	160	164	169
170000	107	121	128	138	148	155	161	163	169
180000	108	119	125	136	146	155	161	164	169
190000	106	121	128	137	146	154	159	162	169
200000	101	119	126	138	146	153	158	161	166

Appendix 2 Table 6. Summary statistics for the simulated earliest time to detection distribution for testing at the border with 100 daily passengers, all times refer to days since the index case

Proportion	centiles								
tested	1st	5th	10th	25th	50th	75th	90th	95th	99th
0.010	118	135	143	159	180	>200	>200	>200	>200
0.020	111	131	140	152	170	189	>200	>200	>200
0.030	103	127	134	148	163	179	196	>200	>200
0.040	108	123	129	142	156	171	186	195	>200
0.050	105	118	126	139	153	165	179	188	>200
0.060	103	117	125	137	150	163	174	182	199
0.070	96	114	122	135	149	161	172	178	191
0.080	97	114	121	134	146	156	167	172	186
0.090	102	115	122	132	144	156	166	172	184
0.100	101	115	122	132	144	154	163	169	180
0.110	98	112	119	130	141	151	161	167	177
0.120	95	112	117	128	140	150	159	164	174
0.130	91	109	116	127	139	148	157	164	172
0.140	97	111	118	128	138	148	157	161	168
0.150	91	110	117	127	137	146	155	161	169
0.160	91	105	114	126	137	146	154	159	168
0.170	97	110	114	125	135	144	153	158	167
0.180	89	105	114	125	134	143	152	157	164
0.190	91	105	114	124	134	143	151	156	164
0.200	87	104	112	123	133	142	150	154	162
0.210	90	103	112	122	132	142	149	155	161
0.220	89	105	111	122	132	141	148	151	158
0.230	88	104	111	122	133	141	148	153	159
0.240	91	104	110	120	132	141	147	152	159
0.250	88	103	110	120	131	140	148	151	157
0.260	83	103	110	121	131	140	146	150	158
0.270	87	103	110	120	130	139	145	150	156
0.280	80	102	111	120	130	138	144	149	154
0.290	86	101	108	119	129	137	144	148	155
0.300	83	100	107	118	128	137	143	148	156
0.310	90	102	110	119	129	137	143	147	153
0.320	87	103	110	118	128	136	143	147	152
0.330	85	101	108	118	127	136	143	146	154
0.340	88	100	108	117	127	136	142	146	154
0.350	83	101	106	116	126	135	141	146	153
0.360	78	100	107	116	126	135	142	145	152
0.370	85	100	107	117	127	135	141	145	153
0.380	82	97	106	116	126	134	141	145	150
0.390	83	97	104	116	125	134	140	144	152
0.400	78	99	106	116	125	133	139	143	150
0.410	78	98	104	115	125	133	140	143	149
0.420	82	99	106	116	125	133	139	144	151
0.430	82	100	106	116	124	133	138	142	148
0.440	77	100	106	115	124	132	139	142	149
0.450	80	97	104	114	124	133	138	142	148
0.460	81	95	103	115	124	131	138	141	148
0.470	87	98	104	114	124	131	138	141	147
0.480	80	97	105	114	123	131	138	141	146
0.490	84	97	104	113	123	131	137	141	149
0.500	84	98	104	113	123	131	136	140	148

Appendix 2 Table 7. Summary statistics for the simulated earliest time to detection distribution for testing at hospitals with seedings from 100 daily passengers

	Proportion	centiles								
IHR	tested	1st	5th	10th	25th	50th	75th	90th	95th	99th
0.005	0.10	135	151	159	170	180	189	196	199	>200
0.005	0.20	125	146	153	164	174	182	188	192	198
0.005	0.30	126	141	149	161	171	179	186	189	194
0.005	0.40	120	139	146	158	167	175	182	186	192
0.005	0.50	118	138	144	156	166	174	180	183	188
0.010	0.10	128	146	152	163	173	183	189	192	197
0.010	0.20	128	140	147	157	167	176	182	186	190
0.010	0.30	120	136	143	153	164	172	178	182	188
0.010	0.40	116	134	141	151	161	169	176	180	186
0.010	0.50	114	132	140	151	160	168	175	178	184
0.015	0.10	126	141	147	159	170	178	185	189	195
0.015	0.20	118	135	143	154	164	173	179	182	187
0.015	0.30	113	133	140	151	161	169	175	178	185
0.015	0.40	112	130	136	147	157	165	173	176	182
0.015	0.50	111	130	136	147	156	164	171	174	180
0.020	0.10	123	139	147	157	167	176	183	186	193
0.020	0.20	118	132	141	151	162	170	176	180	186
0.020	0.30	110	129	136	148	157	166	172	176	182
0.020	0.40	113	127	134	145	155	164	170	173	179
0.020	0.50	106	127	134	144	154	161	168	171	177
0.025	0.10	121	136	144	156	165	173	180	183	189
0.025	0.20	114	131	138	149	159	167	174	177	182
0.025	0.30	114	129	136	146	156	164	171	174	179
0.025	0.40	110	126	133	144	153	162	168	171	175
0.025	0.50	110	124	131	142	151	159	165	169	173

Appendix 2 Table 8. Summary statistics for the simulated earliest time to detection distribution for testing in community surveillance with seedings from 100 daily passengers

Community	Centiles								
survey size	1st	5th	10th	25th	50th	75th	90th	95th	99th
20000	139	156	164	175	185	193	200	>200	>200
30000	132	150	158	170	180	189	195	199	>200
40000	136	151	157	168	178	187	193	197	>200
50000	128	144	154	165	176	184	191	196	>200
60000	129	147	153	164	174	183	190	193	198
70000	124	144	150	163	173	181	189	192	198
80000	125	144	150	162	172	180	186	190	196
90000	122	141	149	160	171	179	185	189	194
100000	128	142	149	159	169	177	184	187	194
110000	123	138	147	158	169	177	183	186	192
120000	123	138	147	158	168	176	183	186	192
130000	125	140	147	157	167	175	182	185	190
140000	124	141	148	158	167	175	182	185	191
150000	121	136	144	156	165	174	180	184	191
160000	119	138	146	156	165	174	180	184	189
170000	116	135	143	153	164	172	179	182	188
180000	118	136	143	154	164	173	178	182	188
190000	118	135	143	154	164	172	177	181	188
200000	117	134	142	153	163	171	178	181	187
